# Cystic partially differentiated nephroblastoma in an 18-month-old girl: a case report

**DOI:** 10.1097/MS9.0000000000001245

**Published:** 2023-09-07

**Authors:** Alia Alhassoun, Albaraa H. Bara, Mohammad N. Ibrahim, Selman M. Berro, Mays G. Khalil

**Affiliations:** a Faculty of Medicine, Aleppo University, Aleppo; b Department of Cardiothoracic Surgery, Damascus University Cardiovascular Surgical Center, Damascus; c Department of Pediatric Surgery, Children’s Hospital; d Department of Otolaryngology, Al-Bassel Hospital, Tartus, Syrian Arab Rebublic; e Peoples’ Friendship University of Russia: Rossijskij universitet druzby narodov, Moscow, Russia

**Keywords:** case report, cystic nephroma, cystic partially differentiated nephroblastoma, nephrectomy, renal tumor, Wilms tumor

## Abstract

**Introduction and importance:**

Cystic partially differentiated nephroblastoma (CPDN) is a rare cystic tumor that affects the kidney. It has a low potential for malignancy. It usually presents as an abdominal mass. It may be difficult to confirm the diagnosis of CPDN without a histopathological study.

**Case presentation:**

The authors report a case of an 18-month-old girl with abdominal distention, which was noticed by her parents. An abdominal computed tomography scan showed a large multilocular cystic mass arising from the lower pole of the left kidney. A left total nephrectomy was performed. Immature blastemal elements without evidence of malignant cells were observed on histological analysis.

**Conclusion:**

The authors report a case of an 18-month-old girl with CPDN managed by total nephrectomy. CPDN should be considered in the differential diagnosis of patients with cystic renal lesions. The authors would also like to affirm that partial or total nephrectomy should be done in all cases of CPDN and other cystic renal tumors.

## Introduction

Highlights

- Cystic partially differentiated nephroblastoma (CPDN) is an extremely rare clinical entity.
- CPDN is a tumor with a low potential for malignancy.
- CPDN commonly presents as an abdominal mass, making it indistinguishable from other kidney tumors.
- Surgery is the treatment of choice with partial or total nephrectomy.
- Local recurrence may occur, while the metastatic disease is not reported.

Cystic partially differentiated nephroblastoma (CPDN) is a rare multilocular cystic tumor that affects the kidney; the incidence of CPDN is ~0.5% according to SIOP (International Society of Pediatric Oncology). It commonly manifests as a unilateral disease, but it may be bilateral; it usually presents before the age of 2^1^. There is a spectrum of multicystic renal tumors, starting with the benign side that includes cystic nephroma (CN), ending with cystic Wilms tumor (CWT); the malignant end of the spectrum. CPDN is located in the middle of this spectrum as a tumor with low potential for malignancy^2^.

CPDN most commonly presents as an abdominal mass, making it indistinguishable from other kidney tumors; it is also difficult to confirm the diagnosis with the radiological study.

On histopathological exam, CPDN is composed entirely of cystic structures separated by septa. While CN contains well-differentiated epithelium without blastemal cells, CPDN contains immature ‘blastemal’ cells within the septal stroma. CWT has a hard element that can be associated with hemorrhage and necrosis^3^.

CPDN is usually a well-circumscribed mass, sharply demarcated from the renal tissue^3^.

Surgery is the treatment of choice with partial or total nephrectomy^4^, local recurrence may occur, while the metastatic disease is not reported. Long-term prognosis after the surgery is very good, to the best of our knowledge.

Here we report a case of CPDN in an 18-month-old girl, managed with total nephrectomy. This work has been reported in line with the SCARE (Surgical CAse REport) criteria^5^.

## Case presentation

An 18-month-old girl with no significant medical or family history was brought to our hospital. Her parents reported that they had noticed abdominal distention 3 weeks prior to the presentation without any other complaint. On initial evaluation, she weighed 11 kg, with generally good condition and normal vital signs. On physical examination, the abdomen was distended, soft, nontender. A palpable, painless, solid, immobile mass occupying the whole abdomen was identified.

Her laboratory tests showed leukocytosis [white blood cell count (WBC)=15 100, neutrophils (*N*)=44%, lymphocytes (*L*)=47%], urinalysis showed 8–10 RBC (red blood cell count)/HPF (high power field), and other laboratory tests, including PT (prothrombin time), PTT (partial thromboplastin time), and BT (bleeding time), were within the normal.

**Figure 1:**
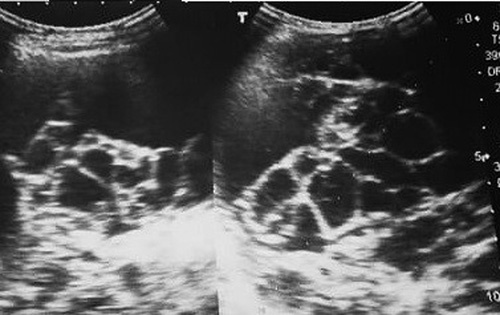
Abdominal ultrasound revealing a well-marginated multicystic mass.

Performing an abdominal ultrasound, we found a well-marginated multicystic mass (Fig. 1).

**Figure 2:**
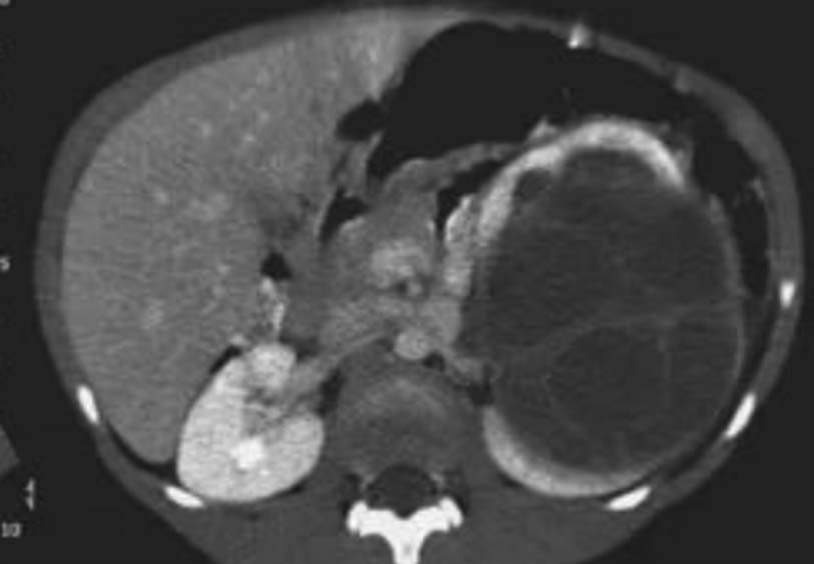
Computed tomography scan, a large multilocular cystic mass arising from the lower pole of the left kidney.

An abdominal computed tomography (CT) scan showed a large multilocular cystic mass arising from the lower pole of the left kidney; it consisted of cystic structures with multiple thick septations between them. No solid components were noticed (Fig. 2).

**Figure 3:**
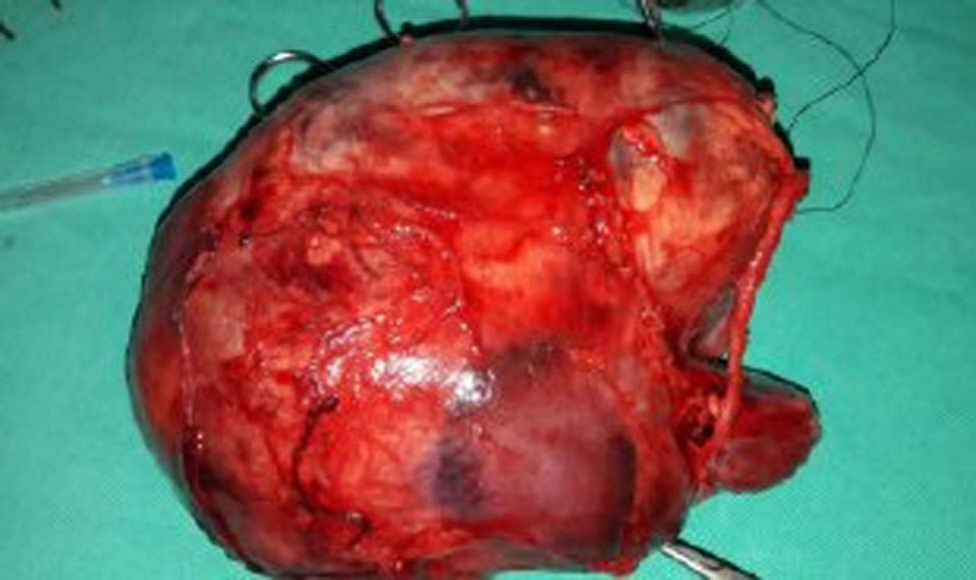
Total nephrectomy was performed; the resected mass measured 15×11×9 cm.

CPDN, cystic standard nephroblastoma (CWT), cystic mesoblastic nephroma (fetal renal hamartoma), and cystic renal cell carcinoma were considered as possible diagnoses. To confirm the diagnosis, we did a left total nephrectomy; the resected mass weighed 800 g and measured 15×11×9 cm (including the kidney and the perirenal fat) (Fig. 3).

**Figure 4:**
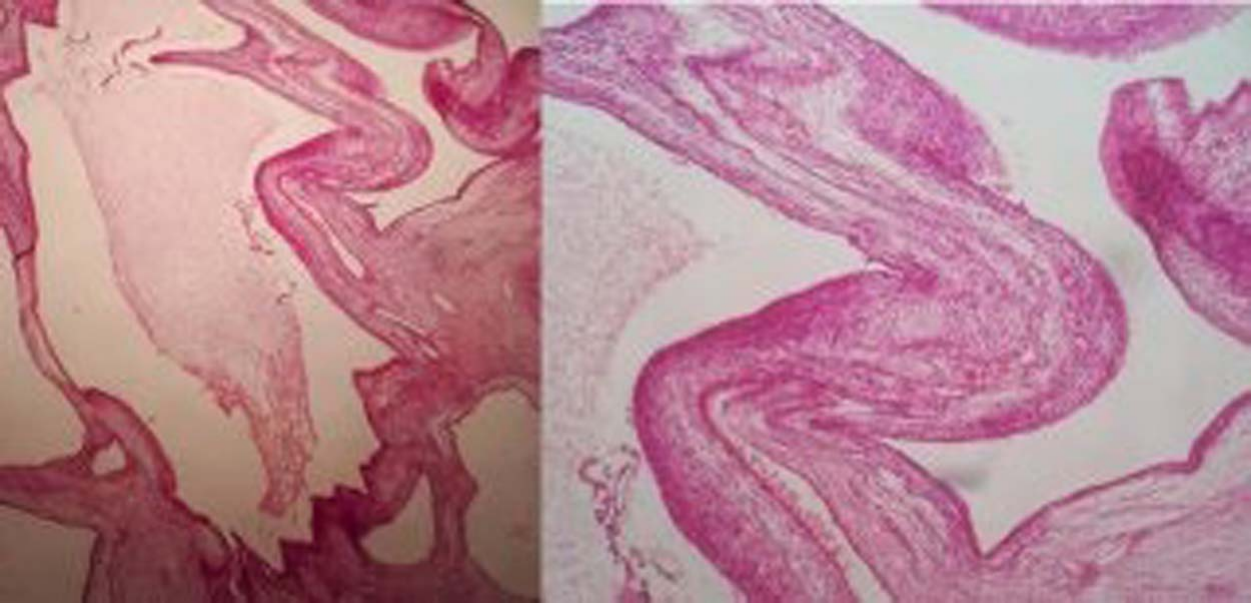
Histological exam, multiple cysts lined by a single layer of flattened to cuboidal low columnar cells with clear cytoplasm, separated by a fibroblastic stroma containing smooth muscle and stratified skeletal muscle fibers, with immature blastematous element.

Histopathological examination (Fig. 4) revealed multiple cysts lined by a single layer of flattened to cuboidal low columnar cells with clear cytoplasm, separated by a fibroblastic stroma containing smooth muscle and stratified skeletal muscle fibers with immature blastematous element. No malignant cells were observed. These findings guide us to the diagnosis of CPDN.

The postoperative period was uneventful. The patient was discharged from the hospital 4 days after the surgery. Two months after the surgery, the patient was in a generally good condition. Four months later, no recurrence was identified on the CT scan.

## Clinical discussion

CPDN is a rare, nonhereditary multiloculated cystic renal tumor, more common in male infants, while there is a predominance incident in the female adult population. Most CPDN cases are diagnosed in early infancy^6^.

Cystic renal tumors were first described in 1892, when Edmonds reported a cystic adenoma; he considered this multilocular cystic lesion as a benign tumor^7^. In 1975, Brown described CPDN as a distinct clinicopathological entity^8^. Later, in 1989, Joshi and Beckwith suggested the main histological features of multilocular cystic renal tumors, outlining the first criteria to distinguish CN from CPDN. According to Joshi and Beckwith, there is a spectrum of disease; the benign side includes CN, a discrete mass with flattened, cuboidal, or hobnail epithelial lining the cyst; the intracystic septa are composed of fibrous tissue that may or may not contain well-differentiated tissues without blastemal cells, while CPDN contains blastemal cells and partially differentiated elements. The malignant side of the spectrum contains CWT, with hard elements, hemorrhage, and necrosis^1^.

CPDN is clinically indistinguishable from other cystic renal lesions. Most renal tumors have nearly the same clinical manifestations, where patients usually present with a palpable mass or visible abdominal distention, even though the majority of them are asymptomatic. The radiological study is also indecisive; on a CT scan, the renal lesion is well-defined, with a pseudocapsule demarcating the lesion from the peripheral renal tissue. Magnetic resonance imaging (MRI) study usually shows cystic compartments with multiple signal intensities, a high signal on MRI may refer to high-density fluids in cystic compartments; MRI cannot differentiate CPDN from other cystic renal tumors^9^.

Partial or total nephrectomy is the treatment of choice, followed by a histopathological exam to confirm the diagnosis of CPDN. Nephron-sparing surgery may be considered, especially in patients with multiple nodules within one kidney or bilateral tumors. In our case, the patient had a large tumor without clear margins to allow a partial resection. For this reason, a total nephrectomy was performed. Preoperative biopsy is not recommended as it may allow tumor cell contamination of the biopsy track^10^. CPDN is not usually associated with lymph node involvement or metastatic disease; it has a very low relapse rate and an excellent outcome after total nephrectomy. Chemotherapy and radiotherapy are generally not recommended for CN and CPDN. In the coming years, genomic study may be helpful to distinguish between CWT, CN, and CPDN; it may even be feasible before surgery by using circulating cell-free DNA techniques^11^.

In our case, the provided CT images are of the best quality we could obtain. Due to the Syrian war, we have some problems with medical records.

## Conclusion

We reported a case of an 18-month-old girl with CPDN, managed by total nephrectomy. We wrote this manuscript to emphasize that CPDN should be considered in the differential diagnosis in patients with cystic renal lesions; we would also like to affirm that partial or total nephrectomy should be done in all cases of CPDN and other cystic renal tumors.

## Ethical approval

No ethical approval was needed.

## Consent

Written informed consent was obtained from the patient’s mother for the publication of this case report and accompanying images. A copy of the written consent is available for review by the Editor-in-Chief of this journal on request.

## Sources of funding

There was no funding.

## Author contribution

A.A.: reviewed the literature and wrote the introduction; M.G.K.: reviewed the literature and wrote the discussion; M.N.I.: reviewed the literature and wrote the case presentation; A.H.B.: checked the spelling and grammar, revised the manuscript, and helped in writing the discussion; S.M.B.: did the histological study and helped in writing the discussion.

## Conflicts of interest disclosure

All authors declare that they have no conflicts of interest.

## Research registration unique identifying number (UIN)

Not applicable.

## Guarantor

Mays G. Khalil, MD.

## Provenance and peer review

Not commissioned, externally peer-reviewed.

## Data availability statement

Not applicable.

## Acknowledgements

The authors thank Hosam Salman, MD; Ahed Hamed, MD; and Riad Cachecho, MD, for their help and support.
